# Health-related quality of life of patients six months poststroke living in the Western Cape, South Africa

**DOI:** 10.4102/ajod.v3i1.126

**Published:** 2014-11-21

**Authors:** Anthea J. Rhoda

**Affiliations:** 1 Department of Physiotherapy, University of the Western Cape, South Africa

## Abstract

**Background:**

The majority of individuals report a decline in health-related quality of life following a stroke. Quality of life and factors predicting quality of life could differ in individuals from lower income countries. The aim of this study was therefore to determine the quality of life and factors influencing quality of life of community-dwelling stroke patients living in low-income, peri-urban areas in the Western Cape, South Africa.

**Method:**

An observational, longitudinal study was used to collect data from a conveniently selected sample of first-ever stroke patients. The Rivermead Motor Assessment Scale and the Barthel Index were used to determine functional outcome and the EQ-5D was used to collect information relating to quality of life at two months and six months poststroke. Descriptive and inferential statistics were used to analyse the data.

**Results:**

The total sample of 100 participants consisted of 50% men and 50% women with a mean age of 61 and a standard deviation of 10.55 years. Six-month quality of life data was analysed for 73 of the 100 participants. Of the 27 who were lost to follow-up, nine participants died, four withdrew from the study after baseline data was collected and eleven could not be followed up as they had either moved or no follow-up telephone numbers were available. A further three participants were excluded from the analysis of the EQ-5D as they were aphasic. Of these, approximately 35% had problems with mobility and self-care, whilst 42% had severe problems with everyday activities and 37.8% expressed having anxiety and depression. Quality of life at two months (*p* = 0.010) and urinary incontinence (*p* = 0.002) were significant predictors of quality of life at six months.

**Conclusion:**

Health-related quality of life was decreased in the South African stroke sample. Functional ability and urinary incontinence were the factors affecting quality of life in the sample. These factors should be considered in the rehabilitation of stroke patients in these settings.

## Background

Stroke is an undisputed major cause of death and disability (Kingley 2011). South African statistics indicate that 60% of stroke survivors are disabled and need assistance with the activities of daily living (Southern African Stroke Prevention Initiative Project Team 2004). This places a major burden on families and communities in South Africa. Patients with stroke may well experience a range of impairments that could impact on physical and psychological functioning, detract from the person’s ability to participate in work and leisure activities, as well as decreasing their quality of life (World Health Organization [WHO] 2001). There is evidence to suggest that patients in the acute stage who are treated in stroke units have better outcomes (Stroke Unit Trialists’ Collaboration 2007). This is not always possible in developing countries like South Africa, due to a lack of resources (Kengne & Anderson 2006); for example, in developing countries, stroke patients are often referred for rehabilitation to primary level outpatient facilities that might not always be the most appropriate settings. The outcomes of stroke, including the quality of life of the individuals, could therefore be nonoptimal due to this lack of access to services.

### Quality of life of stroke survivors

The majority of individuals report a decline in health-related quality of life following a stroke (Hacket *et al*. 2000; Abubakar & Isezuo 2012; Visser-Meily *et al*. 2009). Health-related quality of life is seen as a broad multidimensional construct which includes physical, functional, psychological and social health (WHO 1995). It has been found that all of the above-mentioned dimensions are affected in individuals poststroke (Owolabi 2011), with the emotional and social domains being more affected in certain groups (Visser-Meily *et al*. 2009).

Physical health refers to impairments as a result of the stroke which include motor, speech and sensory difficulties, whilst functional health includes the ability to conduct activities of daily living, mobility and maintain previous life roles. The psychological domain includes the cognitive and emotional challenges experienced by stroke patients. Social support by family and others are included in the social domain (King 1996). In order to fully understand the quality of life of stroke patients, each of these domains needs to be assessed.

A number of factors have been identified which determine the health-related quality of life of individuals after a stroke. Depression and functional status have been repeatedly found to be factors that predict quality of life in stroke survivors (Abubakar & Isezuo 2012; Carod-Artal *et al*. 2000; Howitt *et al*. 2011; Raju, Sarma & Pandian 2010). Stroke severity has also been identified as a factor affecting the quality of life of stroke survivors (Owolabi 2010). Perceived social support (Carod-Artal *et al*. 2000) and level of income (Delcourt *et al*. 2011) have been less frequently reported as factors that could predict quality of life in these individuals.

Data relating to the quality of life in patients with stroke are limited in less resourced countries such as South Africa. As a result of the difference in socioeconomic statuses, differences could be present in the quality of life domains in stroke patients from this low-income country. It has been reported in a South African study that people living in low socioeconomic communities, considered the environmental domain as the most important domain for quality of life (Jelsma, Mkoka & Amosun 2008). Aspects that affected quality of life included access to medical services, owning a brick home (with water, electricity and sanitation) and having sufficient food. The same authors also reported that socioeconomic factors such as income and unemployment were factors that predicted Visual Analogue Scale (VAS) scores as measured by the EQ-5D. It should be noted that there are many other factors that could impact on the quality of life of individuals poststroke. These include factors relating to the stroke such as the severity, cause and side of the lesion in the brain. In addition, personal factors such as the ability of the individual to communicate effectively with others could also impact on their quality of life. Other factors which could impact on the quality of life of individuals poststroke include the use of mobility assistive devices, adaptations made to the physical home environment and input provided by organisations that support individuals and families. However, these factors were not addressed in the current study.

The majority of patients with stroke living in South Africa come from low-income, poorly resourced areas (Rhoda 2012; Rouillard *et al*. 2012) and have limited access to rehabilitation interventions (Kenge & Anderson 2006; Rhoda, Mpofu & De Weerdt 2011). It is not known whether the quality of life domains identified by these stroke patients are similar to those identified in previous populations of stroke patients from developed countries or whether they are similar to those of the general population living in the same environment.

The aim of this study was therefore to determine the quality of life and factors influencing quality of life of community-dwelling stroke patients living in low-income peri-urban areas in the Western Cape, South Africa. This information is important for planning of appropriate, effective rehabilitation services in countries where services for these patients are already limited.

### Materials and methods

This study formed part of a larger study that assessed stroke rehabilitation at Community Health Centres (CHCs). In this research, a longitudinal, observational study design was used to collect the data from a conveniently selected sample of first-ever stroke patients. Data was collected at baseline, two and six-month poststroke intervals.

The study was conducted at state-subsidised CHCs in peri-urban, low-income to middle-income communities in the Western Cape. Community Health Centres are outpatient facilities that offer comprehensive primary healthcare services, which include rehabilitative services. The rehabilitative services include *physiotherapy* and occupational therapy services on a part-time basis and speech therapy offered by students at only one of the centres.

All patients who were consecutively admitted to the centres for therapy and those who met specific inclusion criteria were included in the main study. The inclusion criteria were patients who had experienced a first-ever stroke as defined by WHO (1989), were not more than six weeks poststroke, those who had Rivermead Motor Assessment (RMA) scores of: gross function (RMA-G) ≤ 11; and/or leg and trunk function (RMA-LT) ≤ 8; and/or arm function (RMA-A) ≤ 12, between < 35 and > 85 years of age. Patients who had suffered a previous stroke would be excluded as they could have impairments as a result of the previous stroke. Patients were excluded if they had other neurological impairments with permanent damage, such as previous head injuries or spinal cord injuries, if they had stroke-like symptoms due to subdural haematoma, a brain tumour, encephalitis or trauma, if their stroke had occurred more than six weeks before, a prestroke Barthel Index Score of < 50, and if no informed consent had been obtained from the patient or family. Patients who had suffered a previous stroke were excluded as they could have impairments as a result of the previous stroke which could have impacted on their quality of life and other outcomes.

Before the study commenced, the necessary ethical clearance and permission were obtained. To inform the therapists working at the CHCs about the study, the researcher attended one of the monthly meetings of the CHC therapists at which the researcher presented the study proposal and highlighted the aims, objectives and significance of the study, as well as the ethical considerations that would be adhered to during the implementation of the study. The therapists were informed that they would be contacted on a weekly basis to enquire about new patients with stroke who had suffered a first-ever stroke and who had suffered their stroke not more than six weeks ago. Once the names and contact details of eligible patients were obtained, they were contacted and an appointment was set up. In cases where telephone numbers were not available, the researcher or research assistants went to the patient’s home. The aim of the study was explained to the patient and, in some cases, to the patient’s family or caregiver. The patients were invited to participate in the study and were asked to give written informed consent. In cases where the patient was not able to give written informed consent, a family member was approached. Where written informed consent was obtained, the patients were assessed to see whether they met the inclusion criteria. If the patient was eligible to be included in the study, the necessary baseline questionnaires were completed by the researcher or the research assistant, mostly in the participants’ homes or at the therapy departments at the CHCs. Once the researcher had finished collecting the baseline data, the participants were informed that they would be contacted for an appointment for the two-month and six-month follow-up assessments. These assessments were within a window period of seven working days either before or after the actual two-month or six-month poststroke date. Quality of life data was collected from the participants who were able to verbally provide responses.

### Data collection instruments

Data was collected by using a demographic questionnaire, the EQ-5D for quality of life, the Rivermead Motor Assessment Scale (RMA Scale) and the Barthel Index. Questionnaires for each participant were completed by the same researcher. The EQ-5D is a generic index instrument that focuses on a set of health-related quality of life items to provide a broad assessment. It is a self-administered tool made up of two parts. The first part is designed to obtain an indication of the level of difficulty experienced in mobility, self-care and usual activities. The instrument also assesses the presence and severity of pain and discomfort, as well as anxiety and depression. The second part assesses the individual’s perception of their current health status using a VAS where 0 indicates the worst imaginable health state and 100 the best possible health state (Dorman *et al*. 1998). Good intra-observer agreement (*k* > 0.60) was measured with the use of the EQ-5D in all dimensions measured (Pinto *et al*. 2011). The scale has been used to determine the quality of life of patients in a number of studies that were conducted in South Africa to determine quality of life in patients living with HIV and/or AIDS (Hughes *et al*. 2004), as well as in a local Cape Town community with diverse inhabitants (Jelsma & Ferguson 2004).

The RMA was used to measure motor performance of the participants (Finch *et al*. 2002). The instrument consists of three subscales that measure gross motor function, leg and trunk impairment, as well as arm impairment. The scale is completed by direct observation. The observed activity is scored either as the patient’s ability to do the activity with a score = 1, or the patient’s inability to perform the activity, score = 0. The higher the final score for each subsection, the higher the level of functioning. Finch *et al*. (2002) reported that the RMA is a reliable and valid tool based on good to excellent psychometric properties; for example, excellent scalability coefficients reporting coefficients of scalability (CS), values of gross motor section (*r* = 0.91), leg and trunk section (*r* = 0.81), and arm section (*r* = 0.96). The inter-rater reliability of the scale has also been recorded as being good (Lincoln & Leadbitter 1979). A positive or negative difference of three points of the total on the scale indicates a clinically significant difference.

The Barthel Index was used to determine the level of independence in the basic activities of daily living (Mahoney & Barthel 1965). The Barthel Index consists of 10 items including basic mobility, self-care activities and an assessment of bladder and bowel continence. The items are measured on a graded scale from independence to dependence. The scores range from 0 to 100 with each item being assigned a score of 0, 5, 10 or 15. The Barthel Index can be completed by self-report or by direct observation. An excellent test-retest reliability coefficient *r* = 0.98 has been found for this tool. Shah, Cooper and Maas (1992) reported high correlations between the Barthel Index and the Kenny self-care evaluation (*r* = 0.73). The Barthel Index has been widely used in a number of studies relating to stroke rehabilitation research both internationally (De Wit *et al*. 2007) and locally (Rouillard *et al*. 2012).

### Data analysis

Descriptive statistics were used to analyse the data, which was captured and analysed using the Statistical Package for Social Sciences (version 17). Descriptive frequencies were presented for different quality of life domains. The means and standard deviations were determined for the demographic variables, RMA, Barthel Index and EQ-5D data. Multiple regression analysis was used to determine the factors predicting quality of life at the six-month poststroke interval as determined by the VAS of the EQ-5D. A model was fitted with variables which were found to be significant on univariate analysis (*p* < 0.05). The omnibus test using the enter method was used to determine interaction of the variables. Variables that became non-significant were excluded from the model.

#### Ethical considerations

Ethical approval to conduct the study was granted by the Senate Research Grants and Study Leave Committee at the University of the Western Cape (South Africa). The participants were assured of confidentiality and anonymity and they had the right to withdraw from the study at any stage. There was no obvious risk involved in participating in the study. Patients were referred to the appropriate medical professional at the CHC via the referring therapist if they needed any medical interventions.

## Results

A total of 100 participants were recruited into the study over an 18-month period. Of these, 12 were lost to follow-up at two months, and an additional 12 at the six-month assessment period. Of those who were lost to follow-up, nine participants died, four withdrew from the study after baseline data was collected and 11 could not be followed up as they had either moved or no follow-up telephone numbers were available. No significant differences existed between the participants who dropped out of the study and those who remained in the study (see Table 1). A further three participants were not included in the data analysis of the EQ-5D as they were aphasic. The median time to stroke onset was 21 days (q1; q3 15 days – 31 days).

**TABLE 1:** Mean comparison tests between drop out and nondrop out groups.

Variable	Test	*p*
Age	*t*-test	0.26
Gender	Chi-square	0.61
NIHSS score	Mann-Whitney *U*-test	0.69

NIHSS, National Institute for Health Stroke Scale.

### Sociodemographic status of the participants

The study sample consisted of an equal number of men (50) and women (50). The mean age of the population was 61.0 with a standard deviation (SD) of 10.55 and ages ranged from 36 years to 85 years. A large percentage of the group had a primary school or lower level of education. In addition, a large percentage was unemployed with the majority earning a household income of ≤ R1000.00 per month.

### Functional levels of the participants at six months poststroke

Out of a total score of 100, the participants had a mean Barthel Index Score of 78.6 (SD 23.7) meaning that most were independent in conducting activities of daily living. The means scores for the RMA subscales were as follows:

- for gross motor function, out of a total of 13 the mean score was 9.09 (SD 3.3)
- for lower limb motor function, out of a total of 10 the mean score was 6.66 (SD 2.60)
- for the upper limb motor function, the mean score was 7.53 (SD 4.91) out of a total score of 15.

The results indicate that the participants had better function with regard to gross motor and lower limb function than with upper limb function.

### Quality of life domains

The health-related quality of life of the participants was determined using the EQ-5D. Activities are scored according to a range, from participants having no problem with the activity to participants having a severe problem with performing the activity. The domains of quality of life presented are functional (which includes mobility, activities of daily living and usual activities), physical (which includes pain and discomfort) as well as psychological (which includes anxiety and depression). The results for these domains are illustrated in Table 2. Factors predicting the EQ-5D at six months poststroke are presented in Table 3.

**TABLE 2:** EQ-5D results at six months poststroke (*n* = 73).

Domain	Number (%)		

	No problem	Moderate problem	Severe problem
Mobility	47 (64.4)	23 (31.5)	3 (4.1)
Self-care	47 (64.4)	20 (27.4)	6 (8.2)
Usual activities	15 (20.5)	27 (36.9)	31 (42.4)
Pain and discomfort	37 (50.7)	25 (34.2)	11 (15.1)
Anxiety and depression	46 (63.0)	22 (30.1)	(6.9)

**TABLE 3:** Regression coefficients for factors predicting EQ-5D VAS scores at six months poststroke.

Variable	*B*	SEB	Beta	*t*	Significance
Constant	16.908	15.828	-	1.068	0.289
NIHSS total	0.960	0.952	0.145	1.009	-
Urinary incontinence	-22.765	7.176	-0.353	-3.172	-
Aphasia	12.304	6.809	0.204	1.807	0.076
@2EQ-5D_VAS	0.353	0.133	0.302	2 .662	0.010^*^
@2BItot	0.423	0.247	0.381	1.714	0.091
@2RMA-A	-0.503	0.727	-0.104	-0.691	0.492
@2RMA-LT	-2.292	2.050	0.256	1.118	-
@2RMA-G	-2.241	2.009	0.309	1.115	-

NIHSS, National Institute for Health Stroke Scale; VAS, Visual Analogue Scale; BItot, Barthel Index Total; RMA, A Rivermead Motor Assessment Scale Arm; RMA-LT, Rivermead Motor Assessment Scale Leg and Trunk; RMA-G, Rivermead Motor Assessment Scale Gross Motor Function; SEB, xxx Dependent variable: @6Euro_VAS.

* *p* < 0.05

## Discussion

The aim of the current study was to present the quality of life of individuals with strokes who live in community dwellings. The aspect that was most severely affected in the study was usual activities, which is part of the functional domain of the participants as estimated by the EQ-5D. Quality of life outcomes are best interpreted when compared to a sample of individuals from a similar geographical setting, that is, a sample of nonstroke individuals living in a similar geographical setting. To this end, the findings of the present study were compared to those of previous studies (Hughes *et al*. 2004; Jelsma & Ferguson 2004) conducted in the Western Cape using the same instrument. A larger percentage of participants in the present study (79.5%) experienced difficulty in the domain of usual activities compared with the nonstroke community samples (less than 20%) (Jelsma & Ferguson 2004). A lower percentage (31.7%) of participants living with HIV and/or AIDS in the Western Cape reported having problems with usual activity. This means that stroke patients are experiencing greater challenges in usual activities than those from a similar sample in the community who had not experienced a stroke. It should however be noted that the stroke population was much older than both the community sample mean 50 (SD 17.8) and the sample who had HIV and/or AIDS mean 33.8 (SD 7.8). Even though the stroke patients are older, which could result in a decreased ability to perform usual activities, a similar decreased ability was also reported in a study conducted in a stroke sample in Canada (Pickard *et al*. 2004). It is therefore clear that a stroke impacts on the ability of individuals to continue doing the activities they had previously been doing, which also highlights the role changes that often occur as a result of a stroke (Dowswell *et al*. 2000).

The outcome of this study revealed that usual activity was the category most affected, as measured by the EQ-5D. In the qualitative study conducted at by Rhoda (2012) in South Africa, the majority (61%) of the stroke patients also experienced problems conducting their usual activities. The usual activity domain includes activities such as work and caring for families. This result further supports the finding that the participants had problems with activities that were more difficult to perform than basic activities of daily living and which extended beyond the home environment. These activities could also be classified as part of the participation domain of the International Classification of Functioning Disability and Health (ICF), which is a challenge to patients who have had a stroke (WHO 2001). The results obtained for the mobility construct of the functional domain, using the EQ-5D, correlated well with the functional ability of the participants as determined by the Barthel Index. The mean Barthel score of approximately 79 indicated that most of the participants were able to perform their basic activities of daily living independently (Finch *et al*. 2002).

Pain and discomfort was experienced by a greater number of participants with stroke (49.7%) than the similar community (40.2%) but less than those living with HIV and/or AIDS (80.6%). A larger number of participants in the current study also reported having pain when compared to a stroke sample in the United Kingdom. Although pain has not been identified as a symptom impacting on quality of life, shoulder pain is a common occurrence in patients with stroke which impacts on the functional ability of these individuals (Benlidayi & Basaran 2013).

Thirty-eight percent of participants reported having problems with anxiety and depression, which was similar to the percentage reported in the Woodstock community project (36.4%), and to the HIV sample (31.7%) which was part of the study conducted by Hughes *et al*. (2004). The result for the Woodstock community was high and could be attributed to the high stress levels that may be reported by people from lower socioeconomic parts of the community. These results could suggest that stroke patients living in low-income settings in the Western Cape were not experiencing more anxiety and depression than the general population or those living with HIV and/or AIDS. Depressive symptoms in individuals with stroke should, however, be managed as they impact negatively on functional ability and recovery following a stroke, especially as pharmacological treatment has been found to be effective (Gainotti & Marra 2002). Anxiety and depression have also previously been reported to be related to decreased quality of life in the Netherlands (Visser-Meily *et al*. 2009) and an independent predictor of quality of life in a Nigerian stroke sample (Abubakar & Isezuo 2012; Gibri & Akinpelu 2012). Greater anxiety and depression is significantly correlated with lower physical and psychological health-related quality of life domains in Tanzanian stroke survivors (Howitt *et al*. 2011.). When measured using the anxiety and depression scales, Jones *et al*. (2012) also reported higher levels of depression than what was reported elsewhere.

Activities such as gross motor function and functional activity of the lower limb as measured by the RMA as well as impairments such as urinary incontinent and aphasia were factors that significantly predicted the quality of life two months after a stroke. Urinary incontinence has been identified as a long-term problem following a stroke (Patel *et al*. 2001), which needs to be addressed in rehabilitation of stroke patients.

Functional ability, which is encompassed in the RMA and EQ-5D, is a factor which clearly predicts quality of life in the sample even at the six-month interval. This finding concurs with findings of local, African and international studies (Abubakar & Isezuo 2012; Delcourt *et al*. 2011; Howitt *et al*. 2011; Raju *et al*. 2010). The focus of rehabilitation of patients with stroke should therefore be to improve functional ability in order to improve quality of life of these individuals.

### Limitations

The large number of patients that were lost to follow-up is a limitation of this study. A larger sample size would have allowed an increased number of variables to be included in the regression analysis.

## Conclusion

It can therefore be concluded that patients experienced a challenge in the domain of quality of life specifically relating to the ability to perform their usual activities, which relates to the functional domain of quality of life.

These activities include returning to work and engaging in social activities which are the activities needed for re-integration into the community and society. In addition, factors that predict quality of life in the stroke population studied were similar to those of stroke patients from developed countries.
